# Modulation of inflammatory response related to severe peritonitis by polymyxin-B haemoperfusion

**DOI:** 10.1186/2197-425X-3-S1-A432

**Published:** 2015-10-01

**Authors:** R Coudroy, J-C Lecron, DM Payen, R Robert, and the ABDOMIX Group

**Affiliations:** CHU Poitiers, Service de Réanimation Médicale, Poitiers, France; Université Poitiers, INSERM CIC 1402 (Équipe 5 ALIVE), Poitiers, France; CHU Poitiers, Service Immunologie et Inflammation, Poitiers, France; Laboratoire Inflammation, Tissus Epithéliaux et Cytokines (LITEC-EA 4331), Université Poitiers, Poitiers, France; Department of Anesthesia & Critical Care & SAMU, CHU Lariboisière, Paris, France; Université Paris 7, INSERM U 1160, Paris, France

## Introduction

Although Polymyxin-B hemoperfusion (PMX-HP) is supposed to improve outcomes in patients with sepsis in adsorbing endotoxin and decreasing systemic inflammation, the recent ABDOMIX study did not demonstrate any beneficial effect of PMX-HP in patients with septic shock due to peritonitis [1]. In this context, cytokine clearance induced by PMX-HP is debated.

## Objectives

To assess the influence of PMX-HP on plasma cytokine concentrations and to identify cytokines associated with day 28 mortality.

## Methods

This ancillary study of the ABDOMIX study investigated the impact of two PMX-HP sessions on day 28 mortality in peritonitis-induced septic shock, blood samples were performed within 10 hours post-surgery (P1), at the end of the first hemoperfusion session in the experimental group or 2 hours after P1 in the control group (P2), 24 hours after P1 (P3), and at the end of the second hemoperfusion session in the experimental group or 2 hours after P3 in the control group (P4). Cytokines such as Tumor Necrosis Factor α, Interferon (IFN) γ, Interleukin (IL) 1β, IL-1α, IL-1RA, IL-6, IL-4, IL-10, IL-17A and IL-22 were assessed using magnetic bead-based immunology multiplex assay or enzyme-linked immunosorbent assay.

## Results

Among the 232 patients included in the ABDOMIX study, 19 patients were excluded due to missing sampling and therefore 213 patients (109 in the hemoperfusion group and 104 in the control group) were included in the analysis. Post-operative P1 cytokine concentrations were not different between the 2 groups. Cytokines variation between P1 and P2 were not different between the 2 groups except for IL-1RA and IL-10 which decreased more in the control group than in the hemoperfusion group (p = 0.016 and 0.047, respectively). Cytokines variations between P3 and P4 were not different between the 2 groups except for IL-10 and IL-22 which both decreased in the control group whereas both increased in the hemoperfusion group (p = 0.002 and 0.04, respectively). Using a logistic stepwise regression model, the absence of decrease in IL-10 and IL-22 between P1 and P3 were associated with day 28 mortality independently from Simplified Acute Physiology Score II (p = 0.002 and 0.04, respectively).

## Conclusions

PMX-HP was not associated with a decrease in cytokines concentrations as compared to control group. Higher levels of IL-10 and IL-22 in PMX-HP group may suggest unexpected deleterious interference between these cytokines and polymyxin-B membrane. The absence of decrease in IL-10 or IL-22 blood concentrations within 24 hours after peritonitis-related septic shock was independently associated with day 28 mortality.

## Grant Acknowledgment

Funded by the PHRC interregional 2012 of the French Ministry of Health
